# Hiding in Plain Sight: The Globally Distributed Bacterial Candidate Phylum PAUC34f

**DOI:** 10.3389/fmicb.2020.00376

**Published:** 2020-03-12

**Authors:** Michael L. Chen, Eric D. Becraft, Maria Pachiadaki, Julia M. Brown, Jessica K. Jarett, Josep M. Gasol, Nikolai V. Ravin, Duane P. Moser, Takuro Nunoura, Gerhard J. Herndl, Tanja Woyke, Ramunas Stepanauskas

**Affiliations:** ^1^Bigelow Laboratory for Ocean Sciences, East Boothbay, ME, United States; ^2^Department of Biology, Williams College, Williamstown, MA, United States; ^3^Department of Biology, University of North Alabama, Florence, AL, United States; ^4^Department of Biology, Woods Hole Oceanographic Institution, Woods Hole, MA, United States; ^5^U.S. Department of Energy Joint Genome Institute, Berkeley, CA, United States; ^6^Institut de Ciències del Mar, Consejo Superior de Investigaciones Científicas (CSIC), Barcelona, Spain; ^7^Centre for Marine Ecosystems Research, Edith Cowan University, Joondalup, WA, Australia; ^8^Institute of Bioengineering, Research Center of Biotechnology, Russian Academy of Sciences, Moscow, Russia; ^9^Division of Hydrologic Sciences, Desert Research Institute, Las Vegas, NV, United States; ^10^Research Center for Bioscience and Nanoscience (CeBN), Japan Agency for Marine-Earth Science and Technology (JAMSTEC), Yokosuka, Japan; ^11^Department of Limnology and Bio-Oceanography, University of Vienna, Vienna, Austria; ^12^Department of Marine Microbiology and Biogeochemistry, Royal Netherlands Institute for Sea Research, Utrecht University, Den Burg, Netherlands

**Keywords:** microbial ecology, uncultivated bacteria, microbial genomics, dark ocean, host-association

## Abstract

Bacterial candidate phylum PAUC34f was originally discovered in marine sponges and is widely considered to be composed of sponge symbionts. Here, we report 21 single amplified genomes (SAGs) of PAUC34f from a variety of environments, including the dark ocean, lake sediments, and a terrestrial aquifer. The diverse origins of the SAGs and the results of metagenome fragment recruitment suggest that some PAUC34f lineages represent relatively abundant, free-living cells in environments other than sponge microbiomes, including the deep ocean. Both phylogenetic and biogeographic patterns, as well as genome content analyses suggest that PAUC34f associations with hosts evolved independently multiple times, while free-living lineages of PAUC34f are distinct and relatively abundant in a wide range of environments.

## Introduction

Recent advances in cultivation-independent techniques such as metagenomics and single-cell genomics have enabled the study of the coding potential of many “candidate phyla” – uncultivated microbial lineages that are distinct from well-classified bacterial and archaeal phyla (Rinke et al., 2013; Brown et al., 2015; Castelle et al., 2015; Anantharaman et al., 2016; Hug et al., 2016; Parks et al., 2017). In the absence of cultivation, single-cell genomics enables functional characterization of these candidate phyla by providing genetic blueprints for individual cells (Stepanauskas et al., 2017). Candidate phylum “PAUC34f,” also known as “SAUL” (sponge-associated unclassified lineage), is a deeply branching lineage of bacteria that was first discovered in 16S rRNA sequence surveys of prokaryotic communities associated with marine sponges (Hentschel et al., 2002; Schmitt et al., 2012; Thomas et al., 2016; Pita et al., 2018). Early attempts to classify these 16S rRNA sequences placed them within Deltaproteobacteria (Hentschel et al., 2002), Deferribacteres, Acidobacteria (Webster et al., 2011), or left them unclassified (Montalvo and Hill, 2011). Subsequent phylogenetic analyses of the 16S rRNA sequence (Kamke et al., 2014; Astudillo-García et al., 2017) identified PAUC34f as a sister-clade to the candidate phylum Latescibacteria, a widely distributed, putatively saprophytic group (Youssef et al., 2015; Farag et al., 2017), and distantly related to the candidate phylum Poribacteria, a group that represents a substantial fraction of sponge microbiomes (Taylor et al., 2007a; Schmitt et al., 2012; Kamke et al., 2014; Steinert et al., 2017).

Since the initial discovery of PAUC34f, representatives have repeatedly been found in molecular surveys of marine sponge (*Porifera*) microbiomes from various species, comprising 0.001–20.7% of the identified microbiota (Montalvo and Hill, 2011; Cuvelier et al., 2014; Hardoim and Costa, 2014; Astudillo-García et al., 2017; Jensen et al., 2017; Weigel and Erwin, 2017). Using metagenomic assembly and binning of sponge-associated DNA, two composite genomes (metagenome-assembled genomes, or “MAGs”) of PAUC34f were analyzed recently, significantly expanding our understanding of this group’s phylogeny and metabolic potential. These analyses revealed the potential for degradation of sponge- and algae-derived carbohydrates, phosphate transport and storage, and signatures of host-association, including eukaryotic-like proteins (Astudillo-García et al., 2017).

To date, most of the discussion around PAUC34f has focused on its role in the sponge microbiome, where bacteria are thought to mediate critical biochemical processes (Taylor et al., 2007b; Fan et al., 2013; Webster and Thomas, 2016; Pita et al., 2018). These sponge-associated microbial communities are distinct from those in surrounding seawater and sediment (Thomas et al., 2010, 2016; Reveillaud et al., 2014; Karimi et al., 2017) and differ among sponge species (Webster et al., 2010; Hardoim et al., 2012; Schmitt et al., 2012; Reveillaud et al., 2014; Thomas et al., 2016; Steinert et al., 2017). However, PAUC34f 16S rRNA sequences have also been detected at low abundance in non-sponge environments, including associations with ascidians (Erwin et al., 2014) and corals (Sunagawa et al., 2010; Kimes et al., 2013), tropical stream sediments (Costa et al., 2015), seawater, and marine sediments (Taylor et al., 2013; Thomas et al., 2016; Karimi et al., 2017; Thrash et al., 2017). Recent analyses of two PAUC34f MAGs recovered from the Gulf of Mexico dead zone, coupled with metatranscriptomics, revealed putatively active PAUC34f representatives with evidence of aerobic and facultative aerobic metabolisms, and capacities for complex carbohydrate degradation (Thrash et al., 2017), a similar metabolic profile to PAUC34f MAGs derived from sponges (Astudillo-García et al., 2017).

**FIGURE 1:**
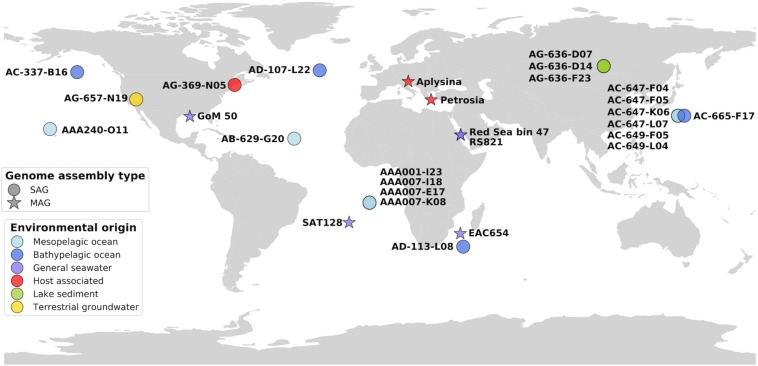
Map of genome origins, colored by sampling environment. SAGs and MAGs are plotted in their approximate geographic areas.

Here we use single-cell genomics, metagenome fragment recruitment, and comparative genomics to investigate the coding potential and global distribution of PAUC34f. We report 21 PAUC34f single amplified genomes (SAGs) originating from the mesopelagic and bathypelagic ocean, lake sediment, terrestrial groundwater, and a tunicate microbiome (Figure 1). We also utilize previously reported MAGs of PAUC34f from various environments: the Red Sea (Delmont et al., 2017; Tully et al., 2018), the northern Gulf of Mexico dead zone (Thrash et al., 2017), the Mediterranean sponges *Aplysina aerophoba* and *Petrosia ficiformis* (Astudillo-García et al., 2017), the East Africa Coast, and the South Atlantic (Tully et al., 2018) (Figure 1). We (i) report the general characteristics of these newly described PAUC34f SAGs and compare them to those of previously analyzed draft genomes; (ii) present an updated 16S rRNA sequence phylogeny, with a focus on the evolution of host-associated, free-living, and other lifestyles; (iii) use a metagenome fragment recruitment approach to assess global environmental abundance, diversity, and specificity of various PAUC34f lineages; and (iv) re-assess putative genomic signatures of host-association, such as eukaryotic-like domains (ELDs) present in PAUC34f lineages from diverse environments.

## Materials and Methods

### Sample Collection

Details of sample collection (dates, locations, and depths) are reported in Table 1. For the marine SAGs, ocean water samples were collected using Niskin bottles. One milliliter aliquots were amended with 5% glycerol and 1× TE buffer (all final concentrations), and stored at −80°C until further analysis. The tunicate sample was immediately placed on ice until processed in the lab. Approximately 100 g of tissue was rinsed, placed in a Ninja blender and homogenized for 10–20 s. The homogenate was passed through a 100 μm filter, amended with 5% glycerol and 1× TE buffer and stored at −80°C.

**TABLE 1:** Assembly statistics and sampling metadata for the 21 PAUC34f SAGs reported in this study.

Genome	IMG genome ID	Cluster	Sampling date	Sampling location	Depth (m)	Assembly size (Mbp)	Estimated completeness (%)	Estimated contamination (%)	Estimated size (Mbp)	GC%
AAA007-I18^∧^	2713897500	I	11/27/2007	South Atlantic subtropical gyre (12°29.69′ S, 4°59.92′ W)	800	0.9	10	0	9.1	60
AAA007-K08^∧^	2639762666	I	11/27/2007	South Atlantic subtropical gyre (12°29.69′ S, 4°59.92′ W)	800	1.2	33	0	3.6	55
AAA240-O11^∧^	2747842434	I	09/09/2009	North Pacific Gyre (22°45′ N, 158°00′ W)	700	0.1	<1	0	NA	54
AC-647-F05^∧^	2713897497	I	12/13/2011	Izu-Ogasawara Trench (29°09′ N, 142°48.12′ E)	505	0.7	24	0	2.9	53
AC-647-L07^∧^	2713897495	I	12/13/2011	Izu-Ogasawara Trench (29°09′ N, 142°48.12′ E)	505	1.3	37	0	3.7	47
AG-636-D07^∧^	3300016441	I	08/26/2014	Lake Baikal sediment, Russia (52°52.95′ N, 107°10.02′ E)	1471	1.3	18	0	6.9	70
AG-636-D14^∧^	3300016600	I	08/26/2014	Lake Baikal sediment, Russia (52°52.95′ N, 107°10.02′ E)	1471	2.2	43	0.1	5.1	70
AG-636-F23^∧^	3300016601	I	08/26/2014	Lake Baikal sediment, Russia (52°52.95′ N, 107°10.02′ E)	1471	2.1	21	0	10.3	66
AG-657-N19^∧^	2747842436	I	12/14/2014	Oasis Valley (NC-GWE-OV-2), Nevada, USA 2 (36°57.6′N, 116°43.2′ W)	9–27	0.3	4	0	7.1	69
AAA007-E17^∧^	2713897501	II	11/27/2007	South Atlantic subtropical gyre (12°29.69′ S, 4°59.92′ W)	800	1.1	37	0	2.9	55
AB-629-G20^∧^	2713897499	II	10/26/2010	Northwestern Atlantic (18°10′ N, 41°2′ W)	511	1	8	0	11.4	53
AC-647-K06^∧^	2713897496	II	12/13/2011	Izu-Ogasawara Trench (29°09′ N, 142°48.12′ E)	505	0.7	19	0.06	3.6	53
AC-665-F17^∧^	2713897492	II	12/11/2011	Izu-Ogasawara Trench (29°09′ N, 142°48.12′ E)	2015	0.5	19	0	2.8	58
AD-113-L08^∧^	2713897490	II	02/18/2011	Indian Ocean (33°33.75′ S, 39°53.07′ E)	4000	1.3	21	0	6.4	56
AG-369-N05^∧^ *	2713897489	II	10/05/2015	Tunicate: Boothbay Harbor, ME, USA (43°51.6′ N, 69°34.8′ W)	1	5.8	78	3.5	7.4	46
AC-337-B16^∧^	2639762669	III	02/11/2013	Northeast Pacific (50° N,145° W)	3000	2.2	40	0	5.6	50
AC-649-F05^∧^	2713897494	III	12/13/2011	Izu-Ogasawara Trench (29°09′ N, 142°48.12′ E)	303	0.8	28	0	3.0	47
AC-649-L04^∧^	2713897493	III	12/13/2011	Izu-Ogasawara Trench (29°09′ N, 142°48.12′ E)	303	0.7	36	2.2	2.0	47
AD-107-L22^∧^	2713897491	III	07/02/2012	Northwestern Atlantic (50°51′ N, 28°51′ W)	2000	0.8	4	0	19.7	50
AAA001-I23^∧^	2639762664	^+^	11/27/2007	South Atlantic subtropical gyre (12°29.69′ S, 4°59.92′ W)	800	0.6	14	0	4.5	50
AC-647-F04^∧^	2713897498	^+^	12/13/2011	Izu-Ogasawara Trench (29°09′ N, 142°48.12′ E)	505	0.6	21	0	3.1	53

Sampling date, geographic locations, and depth, as well as genome assembly size, estimated completeness, and GC content are shown. ^∧^Included in metagenome fragment recruitment. *Included in eukaryotic-like domain analysis. ^+^SAG missing 16S rRNA sequence – no cluster assigned.

Terrestrial groundwater samples (25°C, and pH 7.5) were obtained from a 4-inch nominal PVC-cased monitoring well in Oasis Valley, Nye County, NV, United States (36°57.6′N, 116°43.2′ W, 1,084 m elevation) on 14 December, 2014 (Becraft et al., 2017). The well, NC-GWE-OV-2, was drilled in 2011 and samples an alluvial aquifer (silty sand to gravel) located in the discharge zone of the volcanic Pahute Mesa flow system from a screened interval at 9.1–27.4 m below land surface. Samples were fixed in the same manner as the ocean water samples in the field after discharge of 5,962 L (31 hole volumes) with a Grunfos 30SQ130 submersible pump operated at 25 L.min^–1^.

Surface sediment samples were collected from Lake Baikal, Russia, using a gravity corer on 26 August, 2014, at water depth of 1471 m, coordinates 52°52.95′ N, 107°10.02′ E. The core’s wet sediment sample had a temperature of 3.8°C and a pH of 7.32, measured immediately after bringing the sample onboard the ship. Five grams of the upper sediment sample (0–1 cm below the lake floor) was mixed with 20 mL of sterile 1× PBS buffer in a 50 ml tube. The sample was centrifuged for 30 s at 2,500 *g* to remove large particles. One milliliter of supernatant was placed into a sterile cryovial and mixed with 100 μl GlyTE stock (20 ml 100× TE, pH 8.0, 60 ml sterile deionized water, and 100 ml molecular-grade glycerol) and stored at −80°C.

### Single Amplified Genome (SAG) Generation, Identification, and Sequencing

The generation, identification, sequencing, and *de novo* assembly of SAGs was performed at the Bigelow Laboratory Single Cell Genomics Center.^1^ Cells were separated with fluorescence-activated cell sorting and lysed with KOH as previously described (Stepanauskas and Sieracki, 2007; Rinke et al., 2014; Stepanauskas et al., 2017). In most cases, genomic DNA amplification was done with multiple displacement amplification (MDA) (Dean et al., 2002) while WGA-X (Stepanauskas et al., 2017) was employed on SAGs with identifiers AG-369 and AG-636. The small subunit (SSU) rRNA gene sequences of all SAGs were obtained by PCR amplification and Sanger sequencing (Stepanauskas and Sieracki, 2007; Stepanauskas et al., 2017). Sequences were then classified using SILVA Mod v128 (Quast et al., 2013) implemented in CREST (Lanzén et al., 2012). SAGs identified as PAUC34f were selected for whole genome sequencing, which was performed as previously described (Stepanauskas et al., 2017). Assembly contamination, completeness, and size were estimated using CheckM (Parks et al., 2015).

Genomes were annotated using BlastKOALA and GhostKOALA (Kanehisa et al., 2016) as well as using the Integrated Microbial Genomes database (Chen et al., 2019). ELDs were predicted from amino acid sequences using the EffectiveDB server’s EffectiveELD tool in Protein Mode, with a minimum *Z*-score of 4 for enrichment in pathogenic bacteria (Jehl et al., 2011; Eichinger et al., 2016). Numbers of specific domain annotations were counted by keyword searching. Signal peptides were predicted from amino acid sequences using SignalP 4.1 (Petersen et al., 2011). The input was specified as Gram-negative bacteria that may contain TM regions, using default D-cutoff values.

### Phylogenetic Analyses

16S rRNA sequences were aligned using the SINA alignment software (Pruesse et al., 2012). MEGA7 (Kumar et al., 2016) was used to manually trim alignments and construct maximum likelihood phylogenetic trees using the Kimura 2-parameter Model (Kimura, 1980), a Gamma distribution with invariable sites, and 95% partial deletion for 200 replicate bootstraps. The resulting Newick tree was visualized using iTOL v3 (Letunic and Bork, 2016).

### Metagenome Fragment Recruitment

Recruitment of metagenomic reads was performed using the Burrows-Wheel Aligner (BWA) (Li and Durbin, 2010), as described in Pachiadaki et al. (2017). Ribosomal (rRNA) genes were masked and alignment threshold was set to ≥100 nucleotide overlap and ≥95% nucleotide identity (operational definition of microbial species) (Konstantinidis and Tiedje, 2005; Konstantinidis et al., 2006; Jain et al., 2018). A total of 184 metagenomic datasets (Supplementary Table S3) were used from a variety of environments including the euphotic, mesopelagic and bathypelagic ocean realms, sponges, freshwater lakes, and subsurface aquifer groundwater. The relative abundance of each SAG or MAG’s relatives was determined as the fraction of metagenomic reads mapped per megabase of the reference genome. The heatmap plot was created in R (R Core Team, 2017) using the ggplot2 package (Wickham, 2009).

## Results and Discussion

### General Genome Features

The 21 PAUC34f SAG assemblies ranged 0.3–5.8 Mbp in size, with estimated completeness of 1–78% (average 25%) and no detectable contamination (Table 1). Full genome sizes, estimated from the most complete SAG assemblies, ranged 5–8 Mbps (average of 6.3 Mbp; Table 1). These results are in line with published PAUC34f MAGs, which were estimated to be 2–7 Mbp (Astudillo-García et al., 2017; Delmont et al., 2017; Thrash et al., 2017; Tully et al., 2018). Previous comparative studies have suggested that, on average, sponge-associated bacteria have larger genomes than free-living bacterioplankton, possibly because of adaptations to a variable, nutrient-rich environment and/or enrichment in mobile genetic elements (Giovannoni et al., 2014; Horn et al., 2016). This is in contrast to the streamlining observed in many other bacterial symbionts (Giovannoni et al., 2014). The GC content of PAUC34f SAGs ranged 47–70%, indicating high genomic variability, with SAGs from Lake Baikal sediment and Oasis Valley groundwater on the high end of the reported GC spectrum for known organisms (66–70%).

### Phylogeny and Environmental Specificity

**FIGURE 2:**
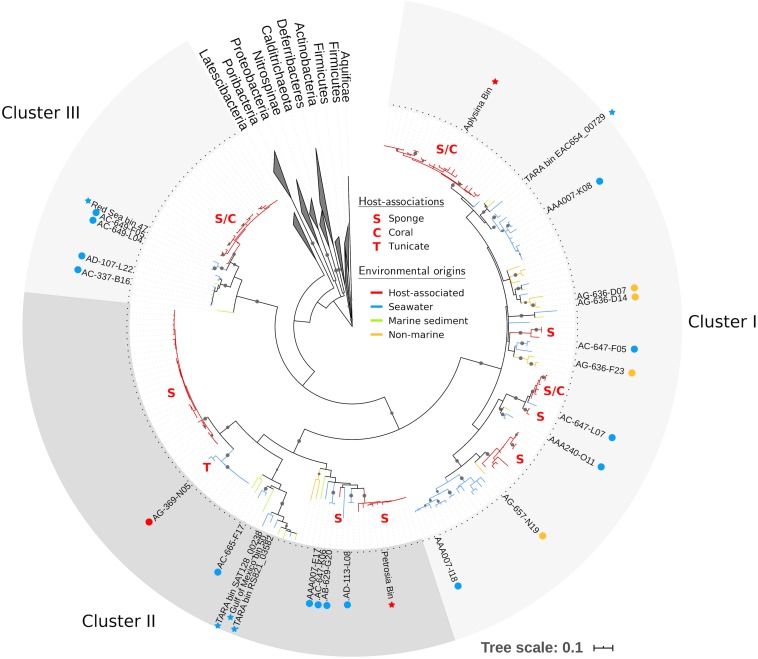
Maximum likelihood tree of PAUC34f using partial 16S rRNA sequences (∼800 bp). Tree includes sequences from PAUC34f genomes (labeled tips) and NCBI sequences with ≥85% similarity to 16S rRNA sequences from PAUC34f genomes (unlabeled tips). Gray circles indicate clades with bootstrap support ≥90%. Branch color indicates the environmental origins of sequences; host-associated clades are additionally annotated with letters indicating the host organism(s) (see Supplementary Table S1 for sequence identifiers and detailed environmental origins). Circles next to labeled tips indicate SAG sequences newly reported here, while stars indicate MAG sequences from other literature – both are colored by environmental origin. Scale bar represents 0.1 nucleotide substitutions per site.

Using our expanded PAUC34f dataset, we revisited phylogenetic analyses of this group. We identified 208 16S rRNA sequences in the NCBI database (as of June 2018) with >85% nucleotide similarity to sequences from PAUC34f SAGs (Supplementary Table S1), an increase from the 93 sequences reported in a previous analysis (Astudillo-García et al., 2017). Phylogenetic analysis of the 16S rRNA sequence resulted in three strongly bootstrap-supported clades (labeled Clusters I, II, and III in Figure 2; cluster labels match those reported in Astudillo-García et al., 2017). The phylogenetic topology was similar to that reported by Kamke et al. (2014) and Astudillo-García et al. (2017).

Of all identified PAUC34f 16S rRNA sequences, 53% were derived from associations with a host: 105 from sponges, 4 from corals, and 1 from a tunicate (Table 2). Notably, 96% of these host-derived sequences clustered together, forming strongly supported (>90% bootstrap), monophyletic clades of at least three host-derived lineages (Figure 2 and Supplementary Table S1). This is in line with the sponge- and coral-specific evolutionary clusters that have been reported across multiple phyla (Simister et al., 2012). The clustering of these sequences suggests the evolutionary distinctness of host-associated and non-host-associated PAUC34f representatives, as reported for two lineages within the candidate phylum Poribacteria (Podell et al., 2018). Notably, lineages from the same host species do not always cluster together, and some host species are distributed across all three major PAUC34f clusters. This suggests multiple, historical environmental acquisitions by hosts throughout the diversification of PAUC34f, supporting the occasional horizontal transfer of organisms proposed for some sponge symbionts (Webster et al., 2010; Schmitt et al., 2012; Reveillaud et al., 2014). However, these are likely historical acquisitions, as most sponge symbionts are host-specific; indeed, some host-associated PAUC34f lineages have been observed to have host-specific vertical transmission (Hardoim et al., 2012; Schmitt et al., 2012; Gloeckner et al., 2013; Reveillaud et al., 2014; Thomas et al., 2016). Interestingly, the tunicate-derived SAG AG-369-N05 did not branch closely with any sponge- or coral-derived sequences. This is in agreement with a recent study demonstrating that ascidian microbiomes share some overlap with those of sponges and corals, but also have distinctive features and high levels of host-specificity (Erwin et al., 2014).

**TABLE 2:** Distribution of 16S rRNA sequences.

		Astudillo-García et al. (2017)	This study
All PAUC34f	Total number of 16S rRNA sequences	93	208
	% host-derived	73.1%	52.9%
	% marine-derived	21.5%	40.4%
	% non-marine derived	5.4%	6.7%
Cluster I	Total number of 16S rRNA sequences	40	99
	% host-derived	65.0%	44.4%
	% marine-derived	25.0%	43.4%
	% non-marine derived	10.0%	12.1%
Cluster II	Total number of 16S rRNA sequences	41	74
	% host-derived	87.8%	59.5%
	% marine-derived	9.8%	37.8%
	% non-marine derived	2.4%	2.7%
Cluster III	Total number of 16S rRNA sequences	12	35
	% host-derived	50.0%	62.9%
	% marine-derived	50.0%	37.1%
	% non-marine derived	0.0%	0.0%

*Environmental origins of PAUC34f 16S rRNA sequences used for phylogenetic tree construction, comparing data from this study to data reported in Figure 3 of Astudillo-García et al. (2017). Environmental origins are defined as follows: host-derived = isolated from a marine sponge, coral or tunicate; marine-derived = isolated from any non-host marine source including seawater, marine sediment, hydrothermal seeps, seafloor lava crusts; non-marine derived = any other source including cave/bioreactor biofilms, hypersaline microbial mats, freshwater, freshwater sediment, groundwater, soil. See Supplementary Table S1 for accession numbers and detailed descriptions of environmental origins.*

Surprisingly, about half of the 16S rRNA sequences derived from this study and public databases originate from non-sponge environments, shifting our largely sponge-focused understanding of PAUC34f (Table 2). Sequences from pelagic environments formed several distinct, bootstrap-supported clusters (Figure 2 and Supplementary Table S1). Environment-specific clades from lake sediments and subsurface aquifers were also found, though less abundant. Notably, Cluster III contained only marine (including marine host) sequences, while Clusters I and II contained 12 and 3% non-marine sequences.

### Global Distribution

In order to further investigate this environmental diversity suggested by the phylogenetic analyses, we used metagenomic fragment recruitment against PAUC34f SAGs and MAGs to assess the distribution and abundance of PAUC34f in various environments. Our main focus was to examine the abundance of PAUC34f in the open ocean, as we recovered multiple SAGs from this environment. Recruitment against Poribacteria and Latescibacteria SAGs and MAGs was also performed for comparative analyses (Table 1). Of the 184 metagenomes used (Supplementary Table S3), 78 were from the *Tara* Oceans Expedition (Pesant et al., 2015), and additional marine metagenomes were from the Malaspina (Duarte, 2015; Acinas et al., 2019) and other oceanographic expeditions, covering all oceanic realms (from the euphotic to the abyssopelagic zone), including oxygen minimum zones (Tsementzi et al., 2016). Additionally, we included metagenomes from environments where PAUC34f SAGs and MAGs have been identified, including the NC-GWE-OV-2 aquifer, Lake Baikal, and sponges.

We also explored the potential role of particles as a niche for the oceanic PAUC34f. The metagenomic datasets from *Tara* and Malaspina were generated from filters of different pore sizes, thus enriched in the “free-living” microbial fraction (0.2–0.8 μm for Malaspina; 0.2–0.8 μm or 0.2–3 μm for *Tara*) or enriched in the “particle-associated” fraction (>0.8 μm for Malaspina; >3 μm for *Tara*) (Pesant et al., 2015; Acinas et al., 2019). However, only Malaspina data included size-fractionated dark ocean metagenomes (Supplementary Table S4); in these data, PAUC34f’s relative recruitment from the free-living fraction was on average seven times higher compared to the particle-associated fraction (Supplementary Figure S1). This difference was statistically significant (*p* = 3.318e-06), implying that the oceanic PAUC34f recovered in our study likely have a predominantly free-living lifestyle. Notably, a published dataset from global Malaspina sites – using 16S rRNA amplicon sequencing – showed PAUC34f to be particularly abundant in the small-particle size fraction (0.8–3.0 μm) in the mesopelagic realm, while being more abundant in the planktonic size fraction (0.2–0.8 μm) in the bathypelagic realm (Supplementary Figure S2; Mestre et al., 2018).

**FIGURE 3:**
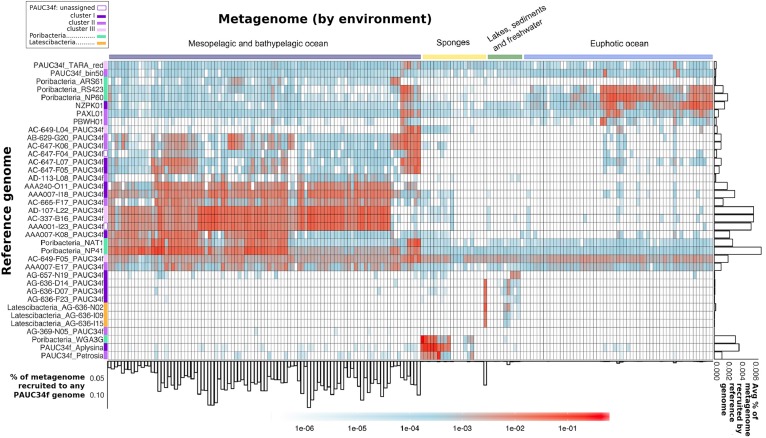
Global distribution of PAUC34f, Poribacteria, and Latescibacteria SAGs and MAGs, as determined by metagenomic fragment recruitment. Reference genomes (SAGs and MAGs) are listed along the *y*-axis, colored by phylogeny. Metagenomes are listed along the *x*-axis and colored by environment (see order and sources of metagenomes in Supplementary Table S3). The heatmap color legend indicates the fraction of metagenome bases recruited per megabase of each reference genome. The bar plot below the *x*-axis indicates the fraction (%) of metagenome bases recruited to at least one of the PAUC34f reference genomes. The bar plot to the right of the *y*-axis shows the average fraction (%) of metagenome bases recruited by each reference genome across metagenomes.

The majority of the PAUC34f SAGs of pelagic origin showed high recruitment of metagenomes from the dark ocean (Figure 3). For these SAGs, the percentage of metagenome bases recruited reached up to 0.14% (Figure 3: see bar-graph on *x*-axis). The same SAGs produced little to no recruitment of metagenomes from the euphotic zone or other environments. The published Malaspina 16S rRNA dataset also reports PAUC34f to be most abundant in the mesopelagic and bathypelagic realms (Supplementary Figure S2; Mestre et al., 2018). In our fragment recruitment, the exceptions are SAGs AC-649-F05 and AAA007-E17, which appear to have cosmopolitan distribution. Although recruitment patterns do not appear to be clade-specific, SAGs with close phylogenetic proximity but different geographical origins have similar distribution patterns (AB-629-G20 from the mesopelagic North Atlantic and AC-647-K06 from the mesopelagic North Pacific recruit similarly, as do AD-107-L22 from the bathypelagic North Atlantic and AC-337-B16 from the bathypelagic North Pacific) (Figure 3). Our observations of recruitment by depth support previous analyses of *Tara* datasets that identified depth (and its associated physicochemical parameters) as a primary driver of community composition (Sunagawa et al., 2015). Within the dark ocean, we observe no apparent dispersal limitation of SAGs between ocean basins, as observed for other dark ocean groups (Pachiadaki et al., 2017). Similarly there aren’t clear distinctions by latitude, as some low-latitude SAGs (AAA007-I18 and AAA001-I23) recruit similarly to high-latitude ones (AC-337-B16 and AD-107-L22) (Figure 3). SAGs from Lake Baikal (PAUC34f: AG-636-D14, AG-636-D07, and AG-636-F23; Latescibacteria: AG-636-N02, AG-636-I09, and AG-636-I15) and from the Oasis Valley subsurface aquifer (PAUC34f: AG-657-N19) recruited highly and specifically from their respective environmental metagenomes.

The sponge-derived MAGs of PAUC34f and Poribacteria recruited almost exclusively sponge metagenomes. Recruitment results corroborate our 16S rRNA sequence results as well as earlier reports indicating that sponge-specific genomes form distinct lineages (Simister et al., 2012). Poribacteria MAGs derived from oceanic metagenomes (“ARS61,” “RS423,” “NP60,” “NAT1,” and “NP41”) recruited largely from open ocean metagenomes and not from sponge metagenomes, supporting their placement within the open ocean-specific *Pelagiporibacteria* lineage (Podell et al., 2018). The tunicate-derived SAG AG-369-N05 did not recruit reads from any metagenomes analyzed in this study; given that no tunicate-derived metagenomes were included in the analysis, this result is unsurprising and supports the organismal specificity observed in ascidian microbiomes (Erwin et al., 2014).

The observed phylogenetic and biogeographic patterns suggest that many PAUC34f lineages are native to the open ocean. Accordingly, a previous metagenomic and metatranscriptomic study suggested that PAUC34f representatives were active in the Gulf of Mexico dead zone (Thrash et al., 2017). Further support for PAUC34f’s role as an important constituent of the dark ocean bacterioplankton comes from a dataset of 2618 SAGs collected from the dark ocean (FASTA included in Supplementary Data Sheet S2). In this dataset, PAUC34f appeared as the ninth most annotated bacterial phylum (out of 18) by 16S rRNA sequences (Supplementary Table S5), again suggesting this group’s non-trivial presence in the dark ocean. Given these results, it is surprising that this candidate phylum has not been reported as a substantial constituent of marine bacterioplankton until now. This may be due to the frequent misclassifications of molecular data (Hentschel et al., 2002; Montalvo and Hill, 2011; Webster et al., 2011). PAUC34f exemplifies significant gaps that may still exist in our understanding of the still-underexplored microbiome of the dark ocean.

### Eukaryotic-Like Domains (ELDs) and Signatures of Host-Association

Having observed PAUC34f’s presence and likely specialization in various non-host environments, we sought to investigate genomic and metabolic features that may facilitate these diverse lifestyles. Previous studies have reconstructed metabolic capabilities in MAGs from the Gulf of Mexico hypoxic zone and from sponges, finding aerobic/facultative aerobic metabolisms, complex carbohydrate degradation, and phosphate storage and transport among other features (Astudillo-García et al., 2017; Thrash et al., 2017). We found little to add to these comprehensive reconstructions, so we focused on assessing potential mechanisms of habitat specialization. Genome-wide annotation profiles revealed no clear partitioning of SAGs and MAGs by phylogeny or by environment (data not shown). Thus, we focused on reassessing genomic features linked to host-association, using the eight most complete PAUC34f genome assemblies (SAGs and MAGs).

Eukaryotic-like domains have been shown to be more abundant in healthy sponge microbiomes compared to the surrounding seawater (Thomas et al., 2010; Fan et al., 2013; Reynolds and Thomas, 2016; Karimi et al., 2017). We sought to assess whether such domains are present in non-sponge-associated representatives as well, and whether they are comparatively enriched within the sponge-associated representatives as potentially adaptive features. Recently, an investigation of two sponge-derived PAUC34f MAGs reported the presence of several of these domains (Astudillo-García et al., 2017). Interestingly, ELDs have also been found in both sponge-associated and free-living Poribacteria (Podell et al., 2018). We found predictions in PAUC34f genomes of several domains that have been reported in sponge microbiome literature (Supplementary Table S6). Numbers of each domain were highly variable among genomes and showed no environmental specificity (Supplementary Table S7).

**FIGURE 4:**
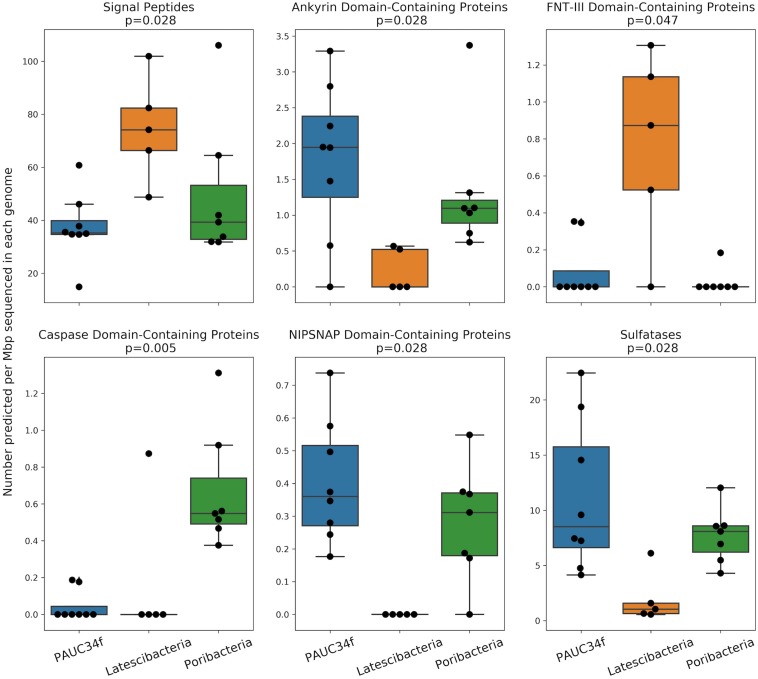
Number of ELDs, ELD-containing proteins, and other sponge-associated features across genomes (SAGs and MAGs) from PAUC34f, Latescibacteria, and Poribacteria. Feature counts are normalized to assembly size (Mbp). Statistics are the results of a Mood’s Median Test (test statistic was Pearson’s chi-squared statistic; values equal to the grand median were counted as below). Only statistically significant comparisons are shown. Remaining comparisons are included in Supplementary Figure S3.

We also compared PAUC34f to two closely related phyla by investigating ELD abundance in SAGs and MAGs of Latescibacteria and Poribacteria (Supplementary Table S2). This comparison is especially interesting because both PAUC34f and Poribacteria consist of many host-associated representatives, while Latescibacteria does not have any known host-association (Farag et al., 2017). Several ELDs showed significant differences in abundance between the three phyla (Figure 4). Of them, Ankyrin and NipSnap were enriched in PAUC34f and Poribacteria, as compared to Latescibacteria, while caspase domains were enriched only in Poribacteria (Supplementary Table S7). Only one domain, FNT3, was enriched in Latescibacteria relative to PAUC34f and Poribacteria.

The *in situ* functions of most ELDs remain elusive, although some domains have been characterized in model systems. Putative functions of these domains include the disruption of phagocytosis (Lee et al., 2002; Nguyen et al., 2014; Reynolds and Thomas, 2016), regulation of gene expression (Viboud and Bliska, 2005), host cell entry (Bierne and Cossart, 2002), cell death, differentiation, signaling, and immune response (Wall and Mccormick, 2014; Huh et al., 2016; Miller et al., 2016), or roles as antioxidants, nutrients, or cofactors (McIntire, 1998; Choi et al., 2008). The FNT3 domain – enriched in Latescibacteria – may facilitate extracellular cellulose degradation (Kataeva et al., 2002), consistent with metabolic analyses of Latescibacteria (Farag et al., 2017). We also found evidence of signal peptides in some of the ELD-containing proteins (Supplementary Table S7), suggesting that these proteins may be active outside the bacterial cell. Notably, the Latescibacteria assemblies were significantly enriched in signal peptides per Mbp (Figure 4). This may reflect the extracellular, cellulosome-affiliated enzymes identified in these bacteria (Farag et al., 2017).

In addition to ELDs, we also examined abundances of several other genome features that have been reported to be enriched in sponge microbiomes, as compared to surrounding seawater and/or sediment, including ABC transporters and sulfatases (Karimi et al., 2017). Neither of these features were more abundant in host-associated vs. free-living PAUC34f genomes (Supplementary Table S5), although PAUC34f and Poribacteria were relatively enriched in sulfatases, as compared to Latescibacteria (Figure 4). Sulfatases are known to facilitate the utilization of sulfated marine polysaccharides (Kamke et al., 2014; Helbert, 2017; Slaby et al., 2017) and are abundant in PAUC34f MAGs from the organic matter-rich Gulf of Mexico hypoxic zone (Thrash et al., 2017). Thus, their presence is not specific to host-associated lifestyle.

These results show the presence of many putatively sponge-related ELDs and other genome features in PAUC34f genomes from various environments. The broad presence of ELDs and sponge-associated features in planktonic lineages of both PAUC34f and Poribacteria (Podell et al., 2018) suggests that these domains may perform different functions in the water column than inside sponge hosts. Seawater contains diverse organic matter-rich microenvironments, such as planktonic cells, detrital particles, and organic gels (Troussellier et al., 2017). Much of the sinking particulate matter in the open ocean is of eukaryotic origin, and ELDs have been suggested as a possible bacterial mechanism to adapt to these microenvironments (Pelve et al., 2017; Podell et al., 2018). The presence of such genome features in ancestral PAUC34f may have facilitated the formation of associations with eukaryotic hosts in some branches of this candidate phylum.

## Conclusion

Our findings suggest a substantial, global presence of PAUC34f in non-host-associated environments, such as freshwater sediments, the terrestrial subsurface, and especially the dark ocean bacterioplankton. Meanwhile, the phylogenetic intermix of host-associated and free-living lineages within this candidate phylum indicates that multiple host acquisition events may have taken place throughout the evolutionary history of PAUC34f, leading to symbiotic associations with sponges, corals, and ascidians. Additionally, genomes from diverse environments contain features often linked with host-association; these features may play adaptive roles in these non-host environments or may have facilitated historical host-acquisition events. Together, our results substantially reframe the environmental context of this enigmatic lineage, indicating that these bacteria could be significant players in diverse environments worldwide.

## Data Availability Statement

All genome data is available in the Joint Genome Institute Integrated Microbial Genome database.

## Author Contributions

MC, EB, and MP contributed to data analysis. RS contributed to data creation and served as the PI of the project. JB, JJ, and TW were scientific correspondents. JG, NR, DM, TN, and GH contributed to sample collection and scientific correspondence.

## Conflict of Interest

The authors declare that the research was conducted in the absence of any commercial or financial relationships that could be construed as a potential conflict of interest. The handling Editor declared a past co-authorship with TW and RS.

## Footnotes

1 http://scgc.bigelow.org
