# Supplementary material for: Hiding in Plain Sight: The Globally Distributed Bacterial Candidate Phylum PAUC34f
Source: Front Microbiol. 2020 Mar 12;11:376. doi: 10.3389/fmicb.2020.00376 (PMC7081726; doi:10.3389/fmicb.2020.00376)
Supplement: Supplementary file 8 [file Data_Sheet_1.docx]

**
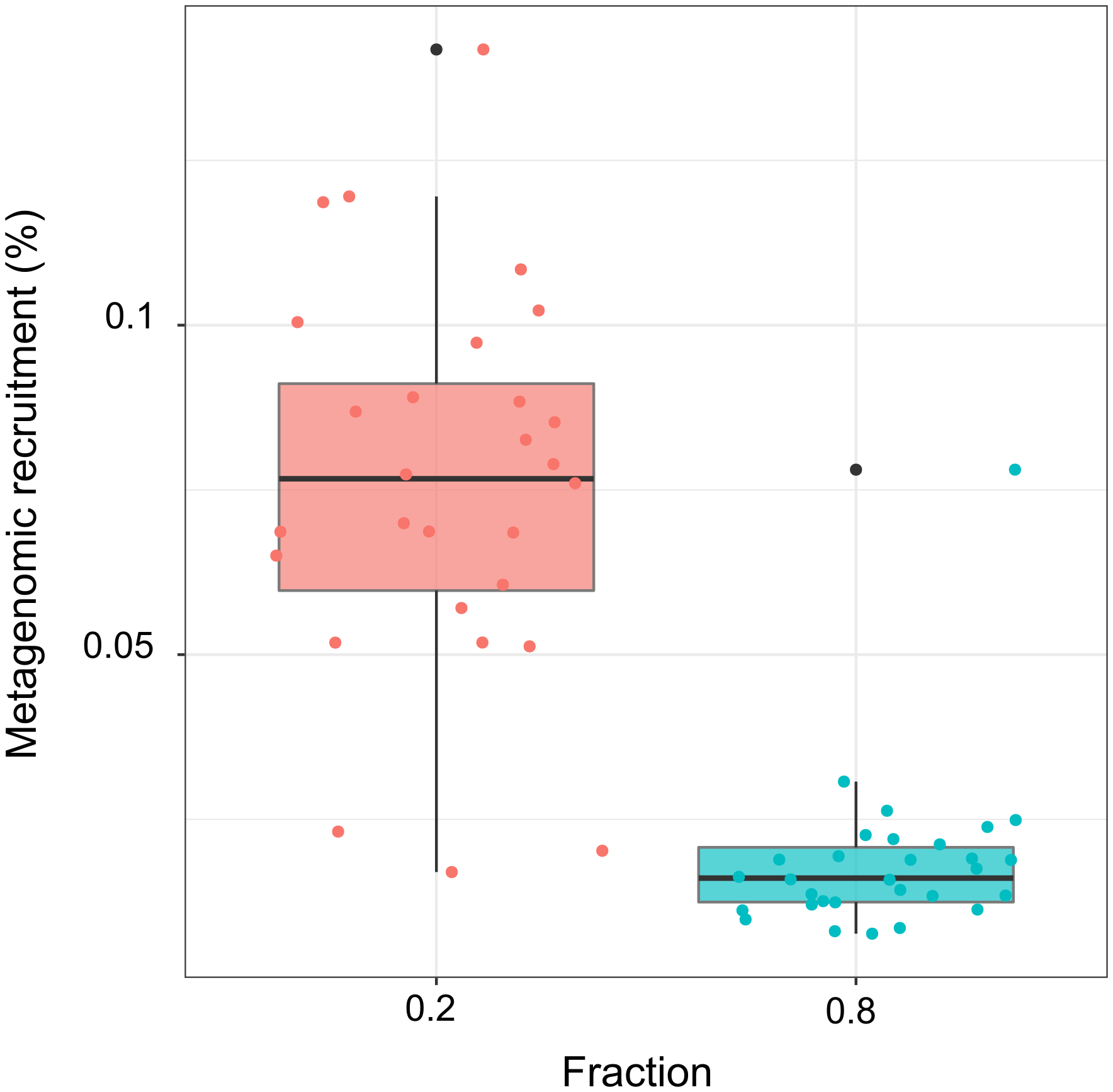
**

**Supporting Information Figure S1:** PAUC34f recruitment of 28 size-fractionated Malaspina metagenomes (Supporting Information Table S4; Acinas et al., 2019). X-axis indicates 0.2 and 0.8 μm fractions. Recruitment was performed using a concatenation of all marine PAUC34f SAGs (Table 1). Each point represents a size-fractionated metagenome, and the y-axis indicates the percent of each metagenome recruited by the PAUC34f SAG concatenation. The difference between the two fractions is statistically significant (Kruskal-Wallis test; p = 3.318e-06).

**
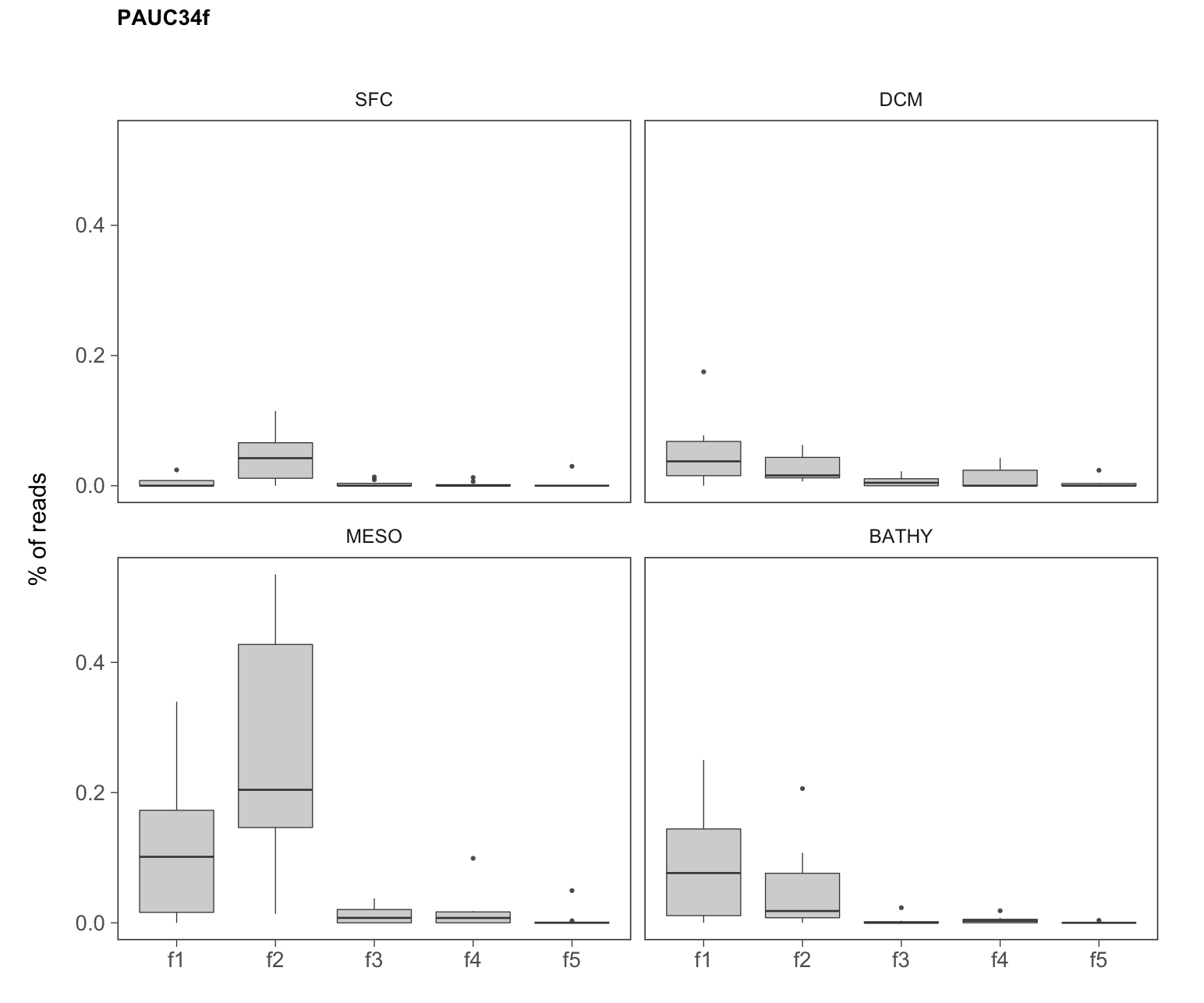
**

**Supporting Information Figure S2:** PAUC34f abundance in the Malaspina 16S dataset from Mestre, *et al.,* 2018. Plots show PAUC34f read abundance in the surface (SFC), deep-chlorophyll maximum (DCM), mesopelagic (MESO), and bathypelagic (BATHY) depth zones. Individual boxes show PAUC34f abundance in various size fractions: 0.2 – 0.8 µm

(f1), 0.8 – 3.0 µm (f2), 3.0-5.0 µm (f3), 5.0-20 µm (f4), and 20-200 µm (f5).

**
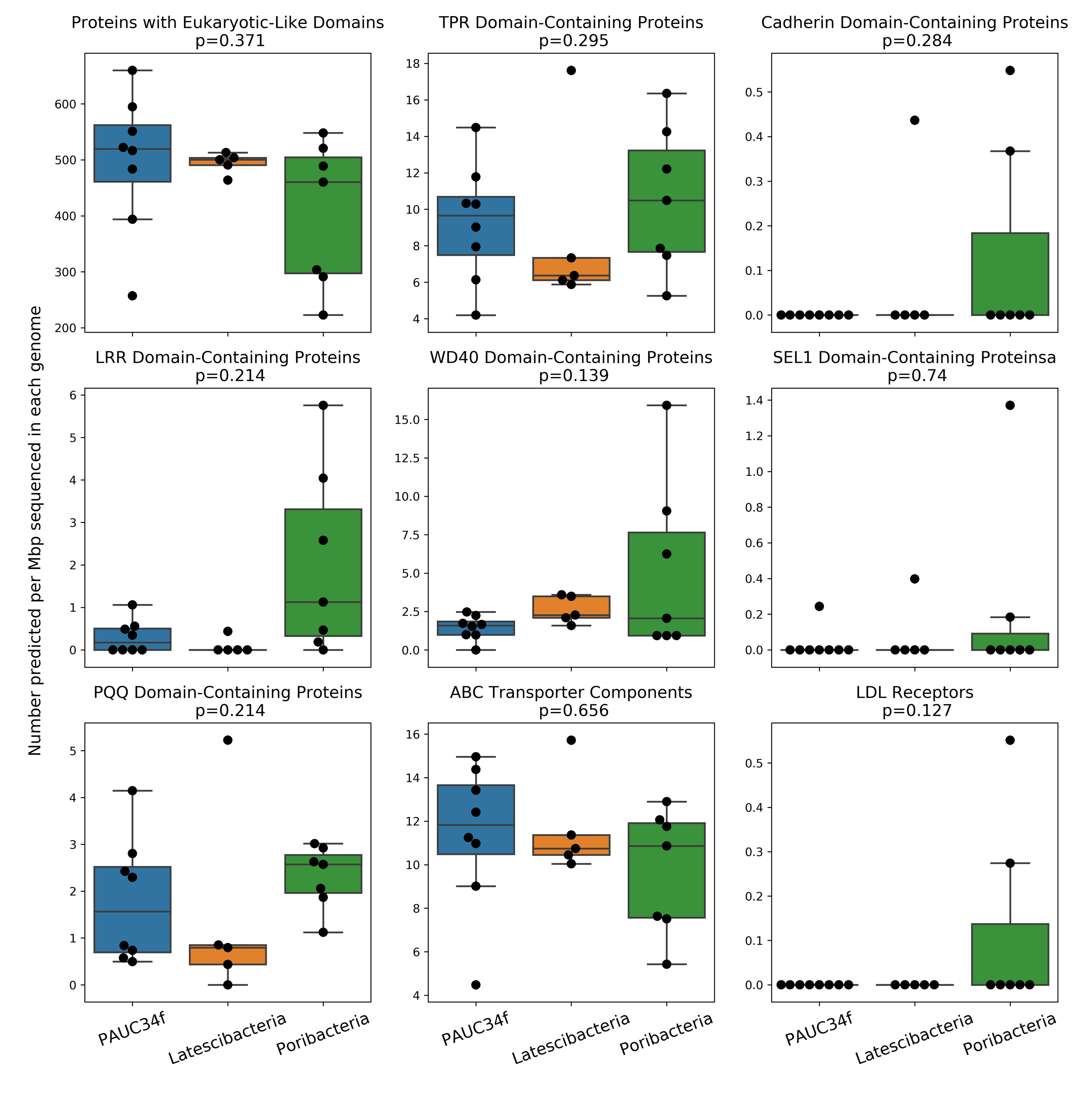
**

**Supporting Information Figure S3:** Number of ELDs, ELD-containing proteins, and other sponge-associated features across genomes (SAGs and MAGs) from PAUC34f, Latescibacteria, and Poribacteria. Feature counts are normalized to assembly size (Mbp). Statistics are the results of a Mood’s Median Test (test statistic was Pearson’s chi-squared statistic; values equal to the grand median were counted as below). Only statistically insignificant comparisons are shown.
